# Vaginal microbiome-hormonal contraceptive interactions associate with the mucosal proteome and HIV acquisition

**DOI:** 10.1371/journal.ppat.1009097

**Published:** 2020-12-23

**Authors:** Laura Noël-Romas, Michelle Perner, Refilwe Molatlhegi, Christina Farr Zuend, Amanda Mabhula, Sarah Hoger, Alana Lamont, Kenzie Birse, Alicia Berard, Stuart McCorrister, Garett Westmacott, Al Leslie, Vanessa Poliquin, Renee Heffron, Lyle R. McKinnon, Adam D. Burgener

**Affiliations:** 1 Departments of Obstetrics & Gynecology, University of Manitoba, Winnipeg, Canada; 2 Center for Global Health and Diseases, Case Western Reserve University, Ohio, United States of America; 3 Medical Microbiology, University of Manitoba, Winnipeg, Canada; 4 Medical Microbiology, University of Kwazulu-Natal, Durban, South Africa; 5 Africa Health Research Institute, Durban, South Africa; 6 Mass Spectrometry and Proteomics Core Facility, National Microbiology Lab, Public Health Agency of Canada, Winnipeg, Canada; 7 Department of Infection and Immunity, University College London, London, United Kingdom; 8 Department of Global Health and Department of Epidemiology, University of Washington, Seattle, Washington, United States of America; 9 Unit of Infectious Diseases, Department of Medicine Solna, Center for Molecular Medicine, Karolinska Institute, Karolinska University Hospital, Stockholm, Sweden; Emory University, UNITED STATES

## Abstract

Alterations to the mucosal environment of the female genital tract, such as genital inflammation, have been associated with increased HIV acquisition in women. As the microbiome and hormonal contraceptives can affect vaginal mucosal immunity, we hypothesized these components may interact in the context of HIV susceptibility. Using previously published microbiome data from 685 women in the CAPRISA-004 trial, we compared relative risk of HIV acquisition in this cohort who were using injectable depot medroxyprogesterone acetate (DMPA), norethisterone enanthate (NET-EN), and combined oral contraceptives (COC). In women who were *Lactobacillus-*dominant, HIV acquisition was 3-fold higher in women using DMPA relative to women using NET-EN or COC (OR: 3.27; 95% CI: 1.24–11.24, *P =* 0.0305). This was not observed in non-*Lactobacillus-*dominant women (OR: 0.95, 95% CI: 0.44–2.15, *P =* 0.895) (interaction *P* = 0.0686). Higher serum MPA levels associated with increased molecular pathways of inflammation in the vaginal mucosal fluid of *Lactobacillus*-dominant women, but no differences were seen in non-*Lactobacillus* dominant women. This study provides data suggesting an interaction between the microbiome, hormonal contraceptives, and HIV susceptibility.

## Author summary

Alterations to the mucosal environment of the female genital tract have been associated with increased HIV acquisition in women. As both the vaginal microbiome and hormonal contraceptives affect mucosal immunity, we investigated their interaction with HIV susceptibility. We characterized the vaginal microbiomes in 685 women from the CAPRISA-004 trial, who utilized three major types of hormonal contraceptives including injectable depot medroxyprogesterone acetate (DMPA), norethisterone enanthate (NET-EN), and combined oral contraceptives (COC). In the 40% of women with *Lactobacillus-*depleted microbiomes, HIV acquisition was not different between contraceptive groups. However, in the 60% of women with *Lactobacillus* as the dominant bacterial taxa, HIV acquisition risk was 3-fold higher (in women using DMPA relative to women using NET-EN and COC). Higher serum medroxyprogesterone acetate levels in *Lactobacillus* dominant women associated with increased cervicovaginal inflammation pathways in the mucosal proteome, biomarkers of which associated with HIV susceptibility. This study provides data suggesting an interaction between the microbiome, hormonal contraceptives, and HIV susceptibility.

## Introduction

The mucosal layer of the female genital tract is the first site of contact for HIV during sexual intercourse. An active immune response in the female genital tract is important to defend against sexually transmitted infections; however, in the case of HIV this can be detrimental, as pre-existing genital inflammation can increase a woman’s risk of acquiring HIV [1, 2]. Thus, factors that can modify the immune landscape in the female genital tract are important for disease susceptibility.

A major contributing factor to the homeostatic balance of the mucosal microenvironment is the microbiome. The vagina contains commensal *Lactobacillus* species that generate lactic acid to lower the vaginal pH, promote epithelial barrier integrity, and increase the secretion of antimicrobial factors [3, 4] to collectively help deter pathogenic organisms. However, the vaginal microflora of many women are instead colonized by anaerobes such as *G*. *vaginalis*, *Atopobium*, *Prevotella*, *Fusobacterium spp*., and others [5, 6], which are often associated with bacterial vaginosis (BV) [7, 8]. Molecular techniques have refined our understanding of the vaginal microbiome, and identified distinct community state-types or cervicotypes that are defined by the dominance of specific taxa [5, 6, 9, 10]. Overall, the prevalence of diverse, *Lactobacillus*-depleted vaginal microbiome profiles are generally higher in studies of women from sub-Saharan Africa [9–12]. These non-optimal vaginal microbiome profiles associate with increased HIV acquisition risk [13], and several microbial taxa have been linked with increased HIV susceptibility [14]. Not surprisingly, such microbiome profiles associate with the production of inflammatory cytokines, epithelial barrier disruption, and the recruitment of HIV target cells [3, 9], indicating that the microbiome impacts the local mucosal immune environment.

Injectable hormonal contraceptives, such as intramuscular depot medroxyprogesterone acetate (DMPA) and norethindrone/norethisterone enanthate (NET-EN) are widely utilized in sub-Saharan Africa, and are more frequently used compared to oral contraceptives, implants, or intrauterine devices. In some studies, DMPA use has been shown to impact the mucosal landscape of the vaginal compartment, which includes an increase in the levels or activation status of vaginal immune cells [15–18]. DMPA has also been linked to increased pro-inflammatory cytokine expression [17, 19, 20]; however, increased cytokine production has not been observed in all studies [16,18,21,22). Animal models have shown DMPA may associate with decreased vaginal epidermal barrier thickness [23–25], which has been confirmed in some human studies [26], but not been in others [15, 27, 28]. Omics analyses of human mucosal samples have suggested DMPA may alter vaginal epithelial repair pathways at a molecular level, which may impact epithelial permeability [29, 30]. While observational studies have linked DMPA usage with increased HIV acquisition risk in women [31–33], the Evidence for Contraceptive Options and HIV Outcomes trial was designed to better assess this question, and showed no significant increase in HIV acquisition risk between DMPA, copper IUD, and LNG implant [34]. While the ECHO trial supported the safety profile of DMPA, interactions between hormonal contraceptives and mucosal immunology are still not fully understood. A recent observational study found an interaction between BV status and injectable hormonal contraceptive use on HIV incidence [35], suggesting mucosal factors may be important.

We previously characterized the vaginal microbiome composition of South African women who participated in the CAPRISA 004 trial [36]. Using these data, which also captures data on host mucosal inflammatory pathways [3, 29, 37, 38], we examined the relationship between the risk of HIV acquisition, the vaginal microbiome, cervicovaginal inflammation pathways, and hormonal contraceptives.

## Results

### Hormonal contraceptive usage in study participants

The use of an effective contraception method was part of the eligibility criteria for the CAPRISA 004 study [39]. Of the 685 participants included in this study, the majority of women were using DMPA (65.6%), while others used either NET-EN (18.0%), or COC (14.2%), at the time of sampling visit (S1 Table). Most women reported using the same contraceptive method at both enrollment and the sample visit (91.7%) (S2 Table). Only 16 women in the trial (2.3%) reported using non-hormonal contraceptives, the majority of which had undergone tubal ligation procedures (n = 13), with the remaining 3 women reporting either having had a hysterectomy, exclusive use of male condoms, or reported using no contraceptives. We had limited statistical power to evaluate the non-hormonal contraceptive group; therefore, this group was included only as a sensitivity analysis in downstream comparisons.

### Vaginal microbial diversity in study participants

We identified bacterial proteins using mass spectrometry analysis of cervicovaginal lavage samples from CAPRISA 004 study participants, which has been published previously in Klatt *et al*. 2017 [36] (see Materials and Methods), and is summarized briefly to describe the metaproteome composition of study participants. Hierarchical clustering was used to distinguished microbiomes into two distinct groups based on predominant bacterial taxa: those who were *Lactobacillus-*dominant (LD) (>50% of bacterial proteins belonging to *Lactobacillus* species) and those non-*Lactobacillus* dominant (non-LD) (S1 Fig). *Lactobacillus* dominant microbiomes had the lowest bacterial taxa diversity (median H-index LD = 0.19), with the majority of *Lactobaicllus* proteins assigned to *L*. *iners* (mean = 61.77%), 18.00% assigned to *L*. *crispatus*, and the remaining 14.52% belonging to other *Lactobacillus* spp. In contrast, those with non-LD profiles were more diverse (median H index = 1.00), where *G*. *vaginalis* was the predominant bacterium (50.40%), with *Prevotella* (7.82%), *Pseudomonas* (5.44%), *Mobiluncus* (5.13%), and other anaerobic taxa (S3 Table). LD women could be separated into two major groups, including those dominated by *L*. *crispatus* (H index = 0.18) and *L*. *iners* (H index = 0.069). In non-LD women two major sub-groups were identified, including those dominated by *G*. *vaginalis* (H index = 0.98) or those that were polymicrobial with higher diversity (H index = 1.16). Baseline clinical, behavioral, and demographic characteristics as well as study arm distribution were similar between LD and non-LD groups (S4 Table). However, a small portion of women with non-LD microbiomes reported using non-hormonal contraceptives methods more frequently than women with LD microbiomes (4.0% vs 1.2%, *P* = 0.0143).

### HIV acquisition risk in LD and non-LD women using different hormonal contraceptives

**Fig 1:**
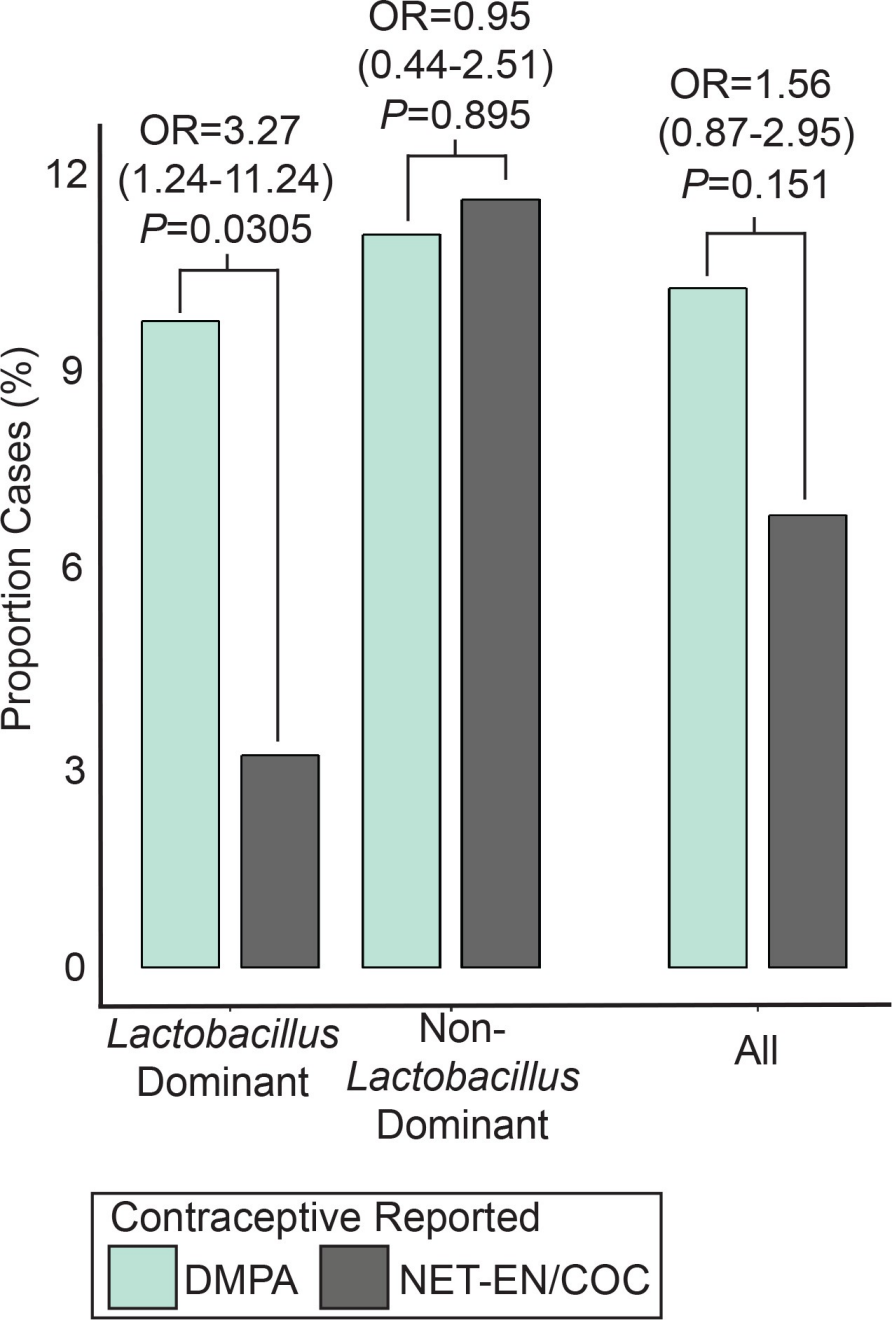
Probability of HIV seroconversion by microbiome type among women using different hormonal contraceptives. Probability of HIV-seroconversion was assessed in women using DMPA relative to other hormonal contraceptives (NET-EN and/or COC) using logistic regression. Comparisons were performed as a stratified analysis of women who had either *Lactobacillus* dominant (n = 407) or non-*Lactobacillus* dominant (n = 287) vaginal microbiome types, or across all participants (n = 685). A significant association between DMPA and HIV-risk was only observed in the subset of *Lactobacillus* dominant women. Full statistical results of the main comparisons can be found in S5 Table.

We assessed the impact of the microbiome on risk of HIV infection among women using different contraceptive types. In the LD group, DMPA use was associated with a 3.27-fold increased probability of HIV infection when compared to women using other hormonal contraceptives (NET-EN or COC) (Frequency of DMPA use: 87.1% [LD Cases] vs 66.5% [LD Controls]; OR: 3.27, 95% CI: 1.24–11.24, *P =* 0.0305) (Fig 1,S5 Table). In contrast, in non-LD women, DMPA was used with similar frequency in cases and controls, (Frequency of DMPA use: 63.3% [non-LD Cases] vs 61.7% [non-LD Controls]; OR: 0.95, 95% CI: 0.44–2.15, *P =* 0.895) (Fig 1, S5 Table). A statistical trend was observed for an interaction between the vaginal microbiome and DMPA use on the probability of HIV acquisition (interaction *P =* 0.0686). The inclusion of non-HC users into the comparison group did not alter findings (S5 Table). Using baseline, rather than visit, data for contraceptive exposure did not change the overall risk estimates associated with DMPA use in either LD or non-LD sub-groups (S6 Table). Adjustments for baseline clinical variables unevenly distributed between DMPA or COC/NET-EN in either microbiome group (S7 Table) did not significantly alter these findings (S8 Table).

The probability of HIV acquisition between LD and non-LD groups was similar (OR: 1.51, 95% CI: 0.89 to 2.57, *P* = 0.123), and adjustments for sexual behaviors (frequency of sex, number of partners, and condom usage), HSV-2 infection, study arm, study site, age and other baseline variables did not significantly alter these findings (S9 Table). Although limited in statistical power, to adjust for any protective effect of *L*. *crispatus*, we analyzed *L*. *iners* dominant women independently, and found the frequency of DMPA use trending higher in cases (83.3% [Cases] vs 66.7% [Controls]; OR: 2.50; 95% CI: 0.91–8.79, *P =* 0.103). We were unable to assess DMPA effects on HIV acquisition in *L*. *crispatus* dominant women as all 3 seroconverters were in the DMPA group.

In an investigation all women in the study, regardless of microbiome type, the association between DMPA and increased HIV acquisition risk was no longer evident when compared to NET-EN and COC as a single reference group (Frequency of DMPA use: 75.4% [Cases] vs 64.6% [Controls]; OR: 1.56, 95% CI: 0.87–2.95, *P* = 0.151), or separately (DMPA vs NET-EN: OR = 1.64, 95% CI = 0.79–3.84, *P* = 0.213; DMPA vs COC: OR = 1.47, 95% CI: 0.68–3.65, *P* = 0.363) (Fig 1, S5 Table). As above, comparing contraceptive use at baseline, rather than at study visit, did not change these observations (S6 Table), and adjustments for baseline variables associated with reported DMPA use did not have a significant impact (S7 and S8 Tables). Overall, DMPA-associated HIV acquisition was only observed in the *Lactobacillus*-dominant group.

### Microbiome interactions with DMPA were not associated with tenofovir gel use and HIV acquisition

As tenofovir was previously reported to show a 39% reduction in HIV acquisition in this cohort [40], we evaluated if the protective effect of tenofovir gel influenced these findings. Study arm was balanced between hormonal contraceptive groups (*P* = 0.6, S1 Table). Adjusted models that included study arm were consistent with the unadjusted results (*P* = 0.164; aOR_LD_ = 3.33 (1.26–11.50), *P* = 0.0285; aOR_non-LD_ = 0.94 (0.43–2.13), *P* = 0.873; aOR_all women_ = 1.54 (0.86–2.92), *P* = 0.164) (S8 Table). Incorporating gel adherence into the adjusted model also did not affect findings (S8 Table). We next investigated the effect of DMPA-microbiome interactions with HIV acquisition risk in women from the placebo arm only (n = 340). Within the placebo arm only, LD women using DMPA were estimated to be at a 3.19-fold increased risk of HIV acquisition, which was similar to the 3.27-fold increase observed when participants randomized to tenofovir were included; however, with the decrease in statistical power, this observation was trending at *P* = 0.0701 (Frequency of DMPA use: 90.5% [Cases] vs 66.5% [Controls]; OR:3.19; 95% CI: 1.04–4.46, *P =* 0.0701). Within non-LD women randomized to placebo, again no effect was observed in non-LD women (Frequency of DMPA use: 68.8% [Cases] vs 67.8% [Controls]; OR:1.05; 95% CI: 0.35–3.51, *P =* 0.939), nor in all women (Frequency of DMPA use: 78.9% [Cases] vs 67.0% [Controls]; OR:1.85; 95% CI: 0.85–4.46, *P =* 0.141). Overall, the 39% protective effect of tenofovir described previously could not account for the hormonal contraceptive-HIV risk findings.

### Bacterial taxa composition in LD and non-LD women using different hormonal contraceptives

We next looked at bacterial composition across hormonal contraceptive groups. The overall composition of the microbiome was similar in women using different hormonal contraceptive methods (S2A and S2B Fig). The DMPA group showed lower alpha-diversity compared to COC and non-hormonal contraceptive groups (median H index = 0.24 (DMPA) vs 0.62 (COC), *P* = 0.0111; Non-HC = 0.98; *P* = 0.0347), but not to NET-EN (S2C Fig). The relative abundance of *L*. *crispatus* and *L*. *iners* was not different between DMPA and either NET-EN, COC or Non-HC users (S2D Fig). Women using DMPA had slightly lower levels of *Gardnerella* (-7.71%, Adj. *P* = 0.0375) and *Megasphaera* (-0.22%, Adj. *P* = 0.0295) compared to the COC users, but there were no differences relative to NET-EN users (S2D and S3A Figs). Importantly, adjusting for these taxa differences did not significantly alter associations between DMPA use and risk of HIV acquisition across the entire cohort (adj. OR: 1.60, 95% CI: 0.89 to 3.04, *P* = 0.129 and adj. OR: 1.53, 95% CI: 0.85 to 2.89, *P* = 0.173 for *Gardnerella* and *Megasphaera* adjusted models, respectively). Further, there were no differences in individual taxa levels associated with hormonal contraceptive use within LD or non-LD women (S3B and S3C Fig, respectively). Therefore, taxa differences between contraceptive groups were minor and could not explain HIV risk associations between contraceptive groups.

### Serum MPA levels associate with cervicovaginal proteome alterations in *Lactobacillus* dominant women

We next examined molecular pathway differences in LD and non-LD women with serum MPA levels. A subset of the cohort (n = 443) had MPA levels quantified by mass spectrometry, which were defined into two groups: those with undetectable or low MPA (≤50–299 pg/ml, n = 253) versus those with moderate to high MPA levels (>300 pg/ml, n = 190), which were denoted “low” or “high” groups for simplicity. Detected MPA levels largely agreed with reported DMPA use, with only 2.87% women who reported DMPA having below detectable MPA levels. Few women who reported using NET-EN, COC or Non-HC had high levels of MPA detected (4.55%, 1.59%, and 0.0%, respectively) (S10 Table). A small number of women from the MPA sub study who reported using either COC or NET-EN at sampling visit had low levels of MPA detected in matched samples (14/85, 16%), of which 8 (9.4%) reported using DMPA at least one visit previously in the trial. No differences were observed in the abundance of individual bacterial taxa according to MPA levels (S11 Table) agreeing with above analyses of microbiome composition by reported contraceptive type.

**Fig 2:**
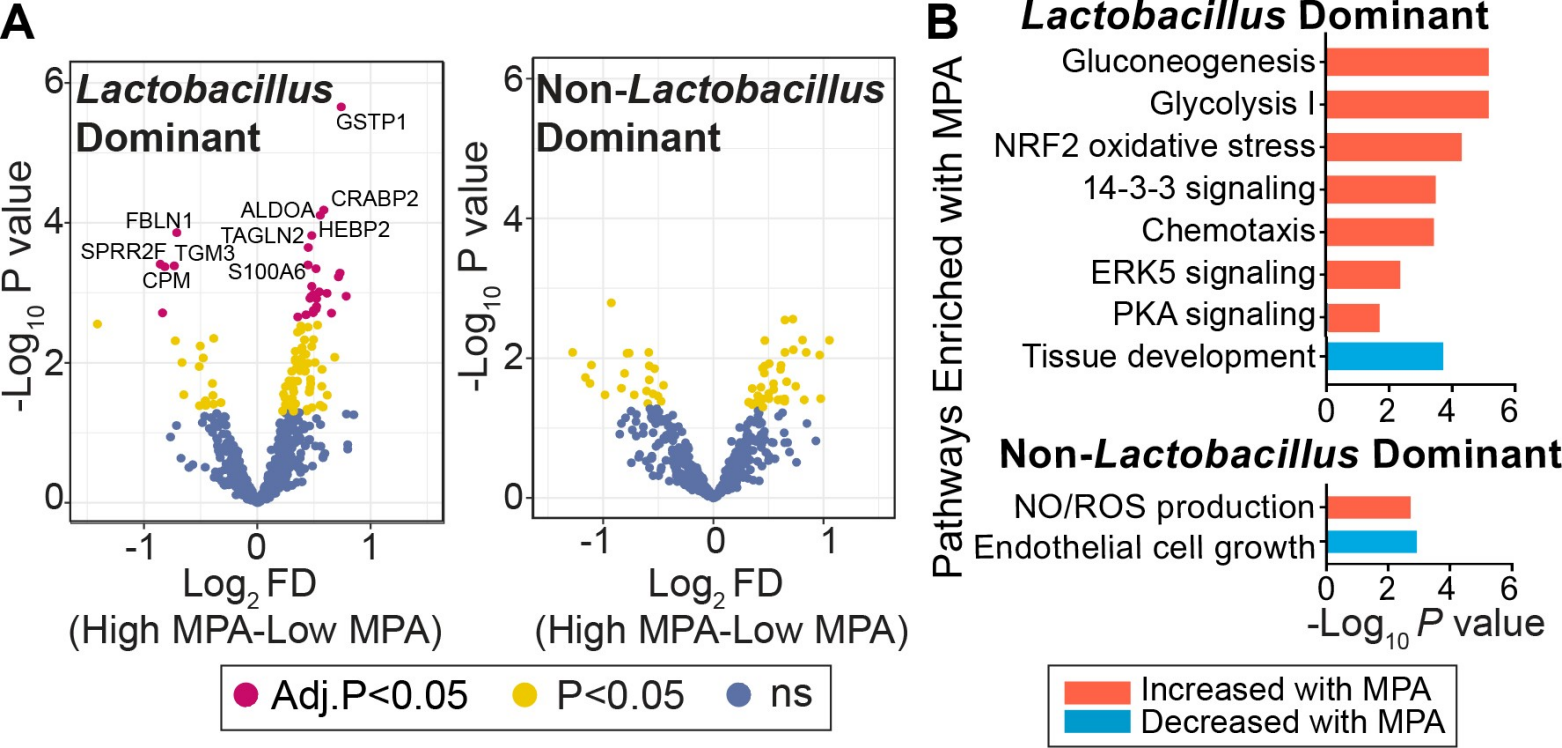
MPA-associated cervicovaginal proteome alterations in *Lactobacillus*-dominant women. (A) Volcano plots display cervicovaginal host proteins differentially abundant in women with “High” MPA (>300 pg/ml, n = 190) vs “Low” MPA (≤50 pg/ml-299 pg/ml, n = 253) for each microbiome type (LD and non-LD) using two-tailed independent t tests (FDR = 5%; 576 proteins; participant numbers: LD-High MPA = 120, LD-Low MPA = 140, non-LD-High MPA = 70, non-LD-Low MPA = 113). Full statistical results and protein descriptions are available in S1 Data. (B) Proteins differentially abundant with MPA levels were annotated to functional pathways using the DAVID Gene Ontology database and the Ingenuity Pathway Analysis database (-log_10_(*P* value) displayed). MPA = Medroxyprogesterone acetate, LD = *Lactobacillus* dominant, non-LD = Non-*Lactobacillus* dominant, ns = non-significant.

Cervicovaginal proteome differences were more pronounced in LD women when stratified by MPA levels relative to non-LD women (S1 Data). A total of 18.2% of proteins were significantly associated with MPA levels in LD women at a stringency of *P*<0.05, and 5% passing an FDR = 0.05, compared to 10.2% of proteins in non-LD women that were significant at *P*<0.05, and none passing the FDR (Fig 2A). These proteome differences in LD women associated with pathways related to glucose metabolism (Glycolysis/Gluconeogenesis), immune activation (ERK, 14-3-3 signaling, cell chemotaxis), the NRF2-mediated oxidative stress response, and tissue development pathways (Fig 2B). Hierarchical cluster analysis of these proteins showed two distinct branches (S4A Fig), where the LD/low MPA group clustered independently from all other groups. Women from the LD/low MPA group were 2.63-fold more likely to be in branch 1 (FET *P =* 1.79E-4, S4B Fig), which associated with lower expression of glucose metabolism and inflammation pathways (S4A Fig). This trend was also observed in *Lactobacillus* subgroups, including *L*. *crispatus* and *L*. *iners* (S4B Fig). In contrast, women from the LD/high MPA group clustered with all non-LD women regardless of MPA level, of which 97.1% were in branch 2, associating with increases in glucose metabolism and inflammation pathways. This relationship was dose dependent between MPA levels and the vaginal proteome. A total of 64/576 proteins (11.1%) correlated with MPA levels in LD women at an FDR = 5%, while no proteins significantly correlated with MPA levels in non-LD women after multiple hypothesis testing correction, which agreed with the “High” vs “Low” MPA analysis presented above (S5 Fig).

**Fig 3:**
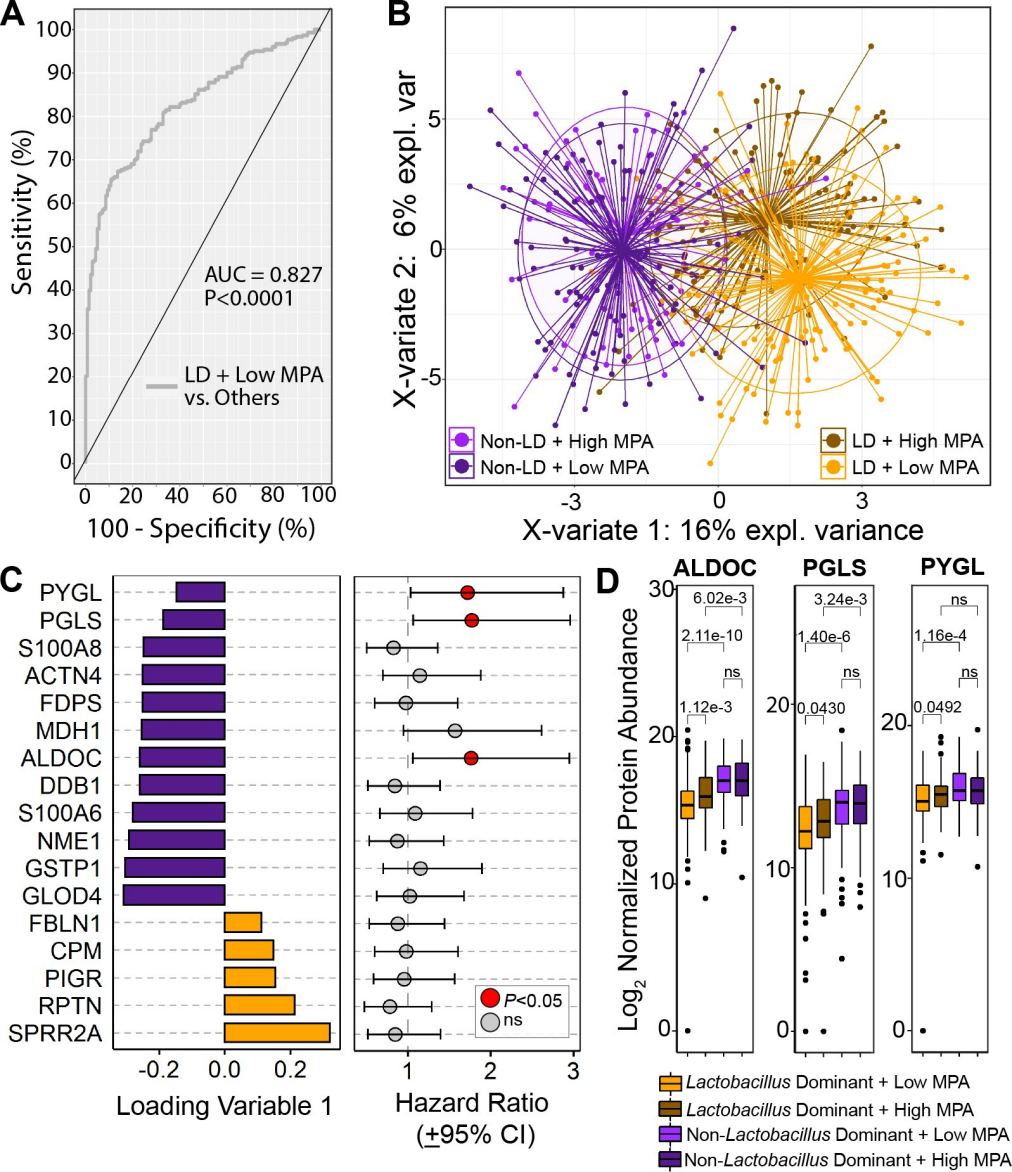
Cervicovaginal protein signatures associated with MPA levels of women with vaginal *Lactobacillus* dominance. A LASSO algorithm identified 17 proteins that best classified LD women with low MPA levels from LD women with high MPA levels, as well as all non-LD women. (A) An area under the curve (AUC) plot shows the protein signature was accurately able to classify women with an LD vaginal microbiome and low serum MPA from other groups (AUC = 0.827, *P*<0.0001). (B) A PLSDA plot shows LD/low MPA women separated from other groups along X-variates 1 and 2. (C) A loadings plot shows protein contributions to the identified signature. Orange bars indicate proteins increased in the LD/low MPA group, while purple bars indicate those increased in all other groups. Box and whisker plots display the hazard ratios (HR) estimated from cox proportional hazards models between MPA-associated glucose metabolism biomarker levels and HIV acquisition risk across all women in the study (n = 685). (D) MPA-risk biomarker expression in microbiome-MPA groupings. LASSO = Least Absolute Shrinkage and Selection Operator, PLSDA = Partial Least Squares Discriminant Analysis, MPA = Medroxyprogesterone acetate, LD = *Lactobacillus* dominant, non-LD = Non-*Lactobacillus* dominant, HR = hazard ratio, CI = 95% confidence interval.

Using a machine learning approach, employing the LASSO (Least Absolute Shrinkage and Selection Operator) method with cross validation by PLSDA (Partial Least-Squares Discriminant Analysis), we identified a minimum set of proteins needed to discriminate these groups. This model showed high classification accuracy (82.7%) (AUC = 0.827, P<0.0001, Fig 3A) to separate women from the LD/low MPA group from those in the LD/high MPA group and all non-LD women (Fig 3B). LASSO identified 17 protein features contributing to this model (Fig 3C, left panel), with 5 proteins positively loaded (higher in the LD/low MPA group) and 12 negatively loaded (higher in all other women: LD/high MPA, and all non-LD women). Positively loaded features associated with epithelial structural functions, including small proline-rich proteins (SPRR2A), repetin (RPTN) and fibulin (FBLN1). Negatively loaded features included detoxification and repair factors (glutathione S transferase P (GSTP1) and DNA damage-binding protein 1 (DDB1)), cell proliferation factors (Nucleoside diphosphate kinase A (NME1)), calcium binding proteins (S100A6/8), and carbohydrate metabolism proteins, such as fructose-bisphosphate aldolase (ALDOC), glycogen phosphorylase (PYGL), and 6-phosphogluconolacotonase (PGLS). Cox proportional hazard analysis showed that having above median levels of ALDOC, PYGL, and PGLS detected in vaginal fluid could predict increased probability of HIV acquisition across all women (Fig 3C, right panel). MPA-risk associated biomarkers showed a gradient effect where abundance was lowest in women from the LD/low MPA group, with significantly increased levels in the LD/high MPA group, which further increased in all non-LD women, where MPA no longer had a significant effect on biomarker expression (Fig 3D and S6 Fig). Overall higher MPA-levels associated with greater degree of altered vaginal mucosal protein levels in LD women that were involved increased carbohydrate metabolism and cellular metabolism, with lower epithelial maintenance biological pathways.

### *Lactobacillus-*depleted vaginal microbiomes associate with molecular signatures of genital inflammation

To better understand why MPA did not associate with proteome differences in non-LD women, we examined microbiome-associated host inflammation pathways. Host proteome alterations were significantly associated with *Lactobacillus* dominance, where 70.8% of the 576 proteins identified (FDR<5%, Log_2_ fold-difference (Log_2_FD) = -3.1 to 1.9) were differentially abundant between women with LD and non-LD microbiome types (S7A Fig). The magnitude of host proteome differences was greatest in women with polymicrobial or *G*. *vaginalis* dominant microbiome profiles in comparison to those with *L*. *crispatus* (58.0% [Log_2_FD: -3.4 to 2.4] and 55.2% [Log_2_FD = - 4.2 to 2.2] significant at FDR = 5%, respectively), while smaller differences were observed between *L*. *iners* dominant group (14.2%, Log_2_FD: - 1.7 to 1.5) (S7A Fig). Having a non-LD microbiome associated with increased host activation of inflammatory pathways (IL-8/IL-6 signaling, leukocyte extravasation signalling), redox regulation pathways (eNOS signaling, NO/ROS production, NRF2-mediated oxidative stress response), carbohydrate metabolism pathways (gluconeogenesis, glycolysis, and the pentose phosphate pathway) and tissue remodeling pathways (integrin signaling, and remodeling of epithelial adherens junctions) in comparison to LD microbiomes (|Z|>0.5, *P*<0.05; S7B Fig). Many functions that were found to be increased in LD women with high serum MPA overlapped with those associated with non-LD microbiomes, including glycolysis I, gluconeogenesis I, protein kinase A signaling, integrin signaling and NRF2-mediated oxidative stress response signalling (S7B Fig).

### Bacterial metabolic pathway differences in women using hormonal contraceptives

Finally, we investigated if bacterial functional pathways were altered in LD women using different hormonal contraceptives. The distribution of major functional pathways did not associate with hormonal contraceptives (S8A Fig). Principal component analysis of KEGG bacterial pathways showed that global functional variation could not be explained by hormonal contraceptive groups (S8B Fig), and no differences were observed in any bacterial pathways in LD women using different hormonal contraceptive types (S8C Fig). Stratification by MPA levels also did not show differences in bacterial functional pathways in LD women.

## Discussion

In this study we showed a relationship between the vaginal microbiome, proteome, hormonal contraceptives, and HIV acquisition risk. Within the subgroup of women with *Lactobacillus-*dominant microbiomes, MPA levels associated with changes in the cervicovaginal proteome and molecular pathways of inflammation. This was not apparent in women with non-*Lactobacillus* microbiota, which exhibited activation of mucosal inflammatory pathways regardless of contraceptive use. These inflammatory signatures associated with >3-fold difference in the probability of HIV acquisition between women on DMPA compared to NET-EN and COC, which was not observed in women with non-*Lactobacillus* dominant communities. While we cannot attribute cause and effect relationship with these features on HIV acquisition in this study, it does suggest that interactions between the microbiome, mucosal inflammation, and hormonal contraceptives are important. Validation of these observations in other cohorts would be an important future direction. While the recent ECHO trial did not observe a significant difference in HIV incidence within women using DMPA, IUDs, or LNG implants [34], examining these mucosal interactions with HIV acquisition during the ECHO trial would be an important avenue of further investigation.

The interaction between DMPA and the mucosal environment are complex. DMPA use associates with alterations to vaginal epithelial pathways and wound healing impairment, as seen in molecular studies of women using DMPA [29, 41]. DMPA use leads to epithelial thinning and an increased proximity of HIV to target cells, which has been observed across several studies [23, 42, 43]. DMPA also has been associated with increased pro-inflammatory cytokines, including IL-6, IL-8, IP-10 and RANTES [19, 44], while others have identified immunosuppressive responses [22, 45], including in a subset of women from the CAPRISA 002 trial [21]. Further, DMPA may suppress antimicrobial defense factors, such as HNP1-3, LL-37 and lactoferrin [46]. Recent data from a mucosal sub-study of the ECHO trial showed an increase in cervical Th17 cells in women after one month of DMPA initiation [47], and other studies report increased cervical HIV target cells [42], which are highly infectible by HIV [48]. These interactions at the mucosal level may be important for HIV susceptibility, and have not yet been fully explored in recent randomized clinical trials evaluating the relative risk of HIV acquisition with different hormonal contraceptives [34].

Consistent with previous studies linking DMPA use and vaginal inflammation, we observed an association of cervicovaginal inflammation pathways with serum MPA levels. These pathways included the oxidative stress response pathways and glucose metabolism. The NRF-2 is a pathway of defense against oxidative stress; downstream effects lead to increased glucose metabolism [49], and this pathway can be influenced by progestins [50]. Indeed, DMPA has been associated with altered glucose metabolism [51] and type-2 diabetes [52]. Glucose metabolism has previously been shown to be important for the recruitment and activation of CD4 + T cells [53], and studies have suggested that glycolysis is an important pathway in the CD4+ T cell immune response [54, 55]. While CD4+ T-cells were not evaluated in this study, these pathways may have relevance for HIV target cell populations in the vaginal mucosa. Further examination of the relationship between these mucosal proteome alterations and HIV target cells would be an important area of future investigation.

Overlapping effects associated with non-*Lactobacillus* microbiomes and DMPA use have previously been observed, including increased levels of pro-inflammatory cytokines such as IL-1, IL-6, and IL-8 [56] and disruptions to the vaginal epithelium [3]. Consistent with this, we observed overlapping activation of pathways between non-*Lactobacillus* microbiomes and increased MPA levels, including increased sugar metabolism (glycolysis), oxidative stress response, immune cell recruitment, tissue remodeling and integrin signaling. It is possible that both DMPA and the microbiome partially converge on similar inflammatory consequences to the genital mucosa. These MPA-associated pathways were more pronounced with *Lactobacillus-*dominant microbiomes. This interaction is consistent with recent publications in CAPRISA 004 showing an effect of the microbiome on cytokine levels and genital inflammation [57].

The influence of hormonal contraceptives on microbiome composition, as well as function, has been reported. In most cases, use of progestin-based hormonal contraception has been associated with a reduction in incidence of BV [32, 58, 59], and higher levels of vaginal *Lactobacillus* spp. [29]; however, others have reported a decrease the levels of vaginal H_2_O_2_-producing lactobacilli [43, 60, 61], increases in microbial diversity [51], or no effect on microbiome composition [62]. We observed only minor differences in microbiome composition and no changes to microbiome-derived functional pathways. As this study focused on bacterial proteome pathways, it is possible differences exist in bacterial metabolic pathways not captured in this analysis, such as the gene or metabolite level. Further, DMPA has also been associated with a higher frequency of other STIs, such as *Chlamydia trachomatis* and *Neisseria Gonorrhoeae* [58, 63]. We saw no differences in STI rates among contraceptive users, nor did we see differences in the levels of *L*. *crispatus* or *L*. *iners*; however, we did observe lower levels of BV-associated bacteria in women on DMPA, such as *Gardnerella* and *Megasphaera*, consistent with other studies mentioned above.

A previous study by Haddad *et al*. utilized Amsel criteria to assess how BV affected HIV acquisition rates in women who used hormonal contraceptives in comparison to non-hormonal contraception [35]. In contrast to our findings, increased HIV risk with DMPA use was observed only in BV+ women. While the study provided important data on longitudinal impacts of DMPA use on HIV acquisition, differences in study design from our analysis may have led to differing results: mucosal data was not collected in this study, non-hormonal contraceptives were included as a comparator, and the vaginal microbiome was classified by clinical BV diagnosis, rather than molecular typing, which can be variable [64]. Differences in genital inflammation between women with vaginal *Lactobacillus* are common, which contributes to increased risk of HIV acquisition, and may have differed in these two study groups. Nevertheless, both of these studies provide supporting evidence that the microbiome interacts with hormonal contraceptives and HIV acquisition and indicate that further studies incorporating other important features, such as genital inflammation, will be an important area of further investigation.

This study has several limitations that must be considered. Primarily, the cross-sectional nature of this study could not assess the impact of microbiome fluctuations or contraceptive switching over time; thus, this study is limited to microbiota and MPA levels at the time of sampling. Studies have shown that the self-reporting of hormonal contraceptive use and sexual behavior can be biased due to difficulties with recall and social desirability, resulting in misclassification [65]. However, less than 3% of women who reported using DMPA had no MPA detected in serum samples, and MPA was detected at high levels in only 1.59% of women using COC and 4.55% using NET-EN. As hormonal contraceptive use was recorded by study personnel, this likely kept misclassification of contraceptive types to a minimum. Also, while condom usage was reported to be lower in women using DMPA, which was included in our adjusted models, we did not observe any significant differences in STI acquisition rates between DMPA users and non-DMPA users. As partner data was not collected, full adjustments for potential HIV exposure could not be assessed. We did not have the ability to evaluate HIV risk in women who did not use hormonal contraceptives, as this was a small group in CAPRISA 004. Further, the contraceptive groupings used in this study were confounded by differences associated with hormonal contraceptive use, including demographic characteristics, age, condom usage, age of sexual debut, and other factors. However, our adjusted models did not show any significant impact on these factors on primary observations. Finally, the size of the contraceptive groups was not balanced, and the majority of women were on DMPA. As CAPRISA 004 was not designed to study this question this limited the statistical power to compare HIV acquisition between different contraceptive groups.

As the primary endpoint of the CAPRISA 004 trial was to determine the effect of tenofovir gel use on HIV acquisition, we took additional steps to evaluate the protective effect of tenofovir on these data. Study arm randomization was balanced between microbiome and contraceptive groupings. Incorporation of study arm and gel adherence into risk models did not alter our findings. A parallel analysis of the effect of DMPA in *Lactobacillus* dominant women randomized to the placebo arm estimated probability of HIV acquisition >3-fold higher relative to other hormonal contraceptives, as was found in the main study. It is important to note that this sub-analysis was reduced in statistical power, and the statistical significance was reduced to *P* = 0.0701. Taken together, our investigation of the 39% protective effect of tenofovir previously reported in Abdool Karim *et al*. could not explain the interaction between DMPA and vaginal microbiome observed in this analysis [40].

Reducing HIV acquisition risk in young women remains a global health priority. This observational study is hypothesis generating and shows that the microbiome, inflammation, and hormonal contraceptives may interact with the risk of HIV acquisition. Thus, consideration of the host-microbiome context is an important consideration for studies of HIV transmission and hormonal contraceptives.

## Materials and methods

### Ethics statement

Ethical approval of this study was provided by the University of Manitoba and University of KwaZulu-Natal’s Human Research ethics committees. Only women who provided written informed consent for storage of their specimens for future research were included in this study (NCT00441298).

### Study design

A total of 685 women from the Centre for the AIDS Programme of Research in South Africa (CAPRISA) 004 trial, who had contraceptive data available, were included in this study. CAPRISA 004 tested the efficacy of a vaginal 1% tenofovir gel for the prevention of HIV infection in women, which have been previously detailed [40]. From the inital 889 women in the trial, 157 women could not be included because of lack of consent to sample storage, 31 samples were technical outliers that fell outside of the normal range of protein signal detected by mass spectrometry, 13 samples did not have bacterial proteins detected, and an additional 3 women declined to report their contraceptive method, which resulted in 23% of this number that could not be included in these analyses.

Cervicovaginal lavage (CVL) samples were collected from study participants during concurrent pelvic examinations at quarterly visits. Sample time points included in this study were those taken from women at the closest time point available prior to seroconversion (mean time prior to seroconversion = 4.7+/-3 months, n = 61), and randomly selected women who did not acquire HIV throughout the trial (n = 624). This post-hoc analysis investigated the effect of contraceptives and vaginal bacterial proteins on host immune pathway expression and HIV acquisition risk. Reported contraceptive method at time of sampling was used to categorize women into two broad categories: Intramuscular depomedroxyprogesterone acetate (DMPA) injection users (n = 449) and those using other forms of hormonal contraceptives, which included 123 women using norethindrone enanthate (NET-EN), and 97 on a combined oral contraceptive (COC). An additional 16 women who used non-hormonal contraceptives (Non-HC) were also investigated.

We hypothesized that *Lactobacillus* levels inferred by mass spectrometry would be an important mediator of hormonal-contraceptive interactions with host immune profiles. To test this, we categorized women into two distinct groups: 1) low immune activation, *Lactobacillus*-dominant profile, and 2) high immune activation, non-*Lactobacillus* dominant profile to assess immune proteome differences associated with hormonal contraceptive use, while performing analysis of specific microbiome groups in sensitivity analysis when possible. Interactions of interest were assessed against HIV outcome in an exporatory case-control analysis using logistic regression as described below. All samples were analyzed by mass spectrometry (MS) to characterize both the human and bacterial proteome at the time of sampling. A complete description of mass spectrometry batch runs, methodology, and data generation parameters are outlined below.

### Sample preparation for mass spectrometry

Details for the preparation of these samples have been published previously [36]. Study samples were randomized prior to processing. Technicians and analysts were blinded to patient identification, HIV seroconversion status and contraceptive method. Equal amounts (25 μg) of proteins from each sample were digested into peptides using filter-aided sample preparation (FASP). Proteins were first denatured in urea exchange buffer (UEB): 8 M Urea (GE HealthCare, Chicago, IL), 50 mM HEPES buffer (Sigma, St. Louis, MO) at a pH = 8.0. Denatured proteins were added to a 10 kDa Nanosep filter cartridge (Pall, Port Washington, NY), and centrifuged at 10,000 x g. Proteins were then reduced with 25 mM dithiothreitol (Sigma) for 20 min at room temperature, alkylated with 50 mM iodoacetamide (Sigma) for 20 min in the dark, and washed in UEB and 50 mM HEPES buffer. Proteins were digested with 2 μg of Trypsin (Promega, Madison, WI) at 37°C overnight. Peptides were eluted off the filter with 50 mM HEPES buffer, dried using vacuum centrifugation, and were cleaned of salts and detergents using reverse-phase liquid chromatography (high pH RP, Agilent 1200 series micro-flow pump, Water XBridge column) in a step-function gradient.

### Mass spectrometry analysis of cervicovaginal mucosa

Mass spectrometry analysis was performed as described previously [36]. Briefly, 1 μg of peptide from each sample were analyzed on a nano-flow Easy 1000 in line to a Q-Exactive Plus mass spectrometer with a nano-electrospray ion source at 2.0 kV (Thermo Fischer Scientific, Waltham, MA), using a 100 C_18_-reversed phase Easy-Spray column ES803 (50 cm length, 100 um diameter, 1.8 um particle; Thermo Fisher Scientific). MS1 survey scans were performed using an Orbitrap ion trap mass analyzer (*m/z* range: 300–1700, resolution: 70,000 at a *m/z* of 200, ACG target: 3e^6^, maximum injection time: 80 ms). Data-dependent spectra acquisition selected the 15 most abundant precursor ions from each survey scan that met isolation width (3 *m/z*), intensity (1e^5^ ions) and charge (+2–5) criteria. Precursor ions were fragmented by high energy collision-induced dissociation (HCD; 28% normalized collision energy). Fragment ion MS2 scans were acquired on the Orbitrap analyzer (dynamic *m/z* range, resolution: 17,500 at a *m/z* of 200, ACG target: 2e^5^, maximum injection time: 100 ms). Reference samples consisting of a pooled CVL sample were run every 10 samples to monitor MS consistency, and were used to assess downstream normalization and data quality. Samples with a median protein signal greater than 1.5 times the interquartile range were determined to be outliers and removed from downstream analysis.

### Proteomics data processing

Metaproteome data was acquired as previously described [36]. Briefly, peptides were searched using the Mascot search engine (v2.4, Matrix Science, Boston, MA) against a curated database, which included the following bacterial genera: *Lactobacillus*, *Gardnerella*, *Pseudomonas*, *Mobiluncus*, *Ruminococcus*, *Prevotella*, *Ruegeria*, *Bifidobacterium*, *Megasphaera*, *Pedobacter*, *Streptococcus*, *Escherichia*, *Atopobium*, *Dialister*, *Fusobacterium*, *Peptoniphilus*, *Peptostreptococcus*, *Porphyromonas*, *Shuttleworthia* and *Sneathia* (UniProt SwissProtKB/TrEMBL, 2015). The database also included human proteins from SwissProtKB (2012) to limit potential homologous identifications between human and bacterial proteins measured. Search results were analyzed using Scaffold Q+ for proteomics (Portland, OR). Bacterial identifications were restricted to those that passed a critical threshold of ≤1% FDR protein at the protein level, ≤0.1% FDR at the peptide level, and had ≥2 unique peptides identified per protein. Scaffold accession reports containing protein homology information used to identify proteins that matched to more than one genus or species. Proteins ambiguous at the species level were binned to the bacterial genus, and proteins that were ambiguous at the genus level were binned into an ‘undistinguishable’ category. Protein abundances were quantified by spectral counting, and microbial taxa abundance was estimated by the summed spectral counts of all proteins uniquely assigned to that genus or species. Women with >50% of all bacterial proteins classified to the *Lactobacillus* genus, were categorized as “*Lactobacillus* dominant” (LD), women with ≤50% of bacterial proteins attributed to *Lactobacillus* were categorized as non-*Lactobacillus* dominant (non-LD). Differences in the relative abundance of bacterial taxa levels between contraceptive groups were evaluated by Man-Whitney U tests with adjustments for multiple hypothesis testing using the Bonferroni method (FDR = 5%). Bacterial proteins were functionally annotated by KEGG gene ontology. Bacterial functional pathway composition was analyzed by Fishers exact tests (*P*<0.05), categorizing pathways by either above below median expression across the cohort, which are reported in the manuscript in R (v.3.5.1).

For human proteins, MS spectra were processed by Progenesis QI (v21.38.1432, Nonlinear Dynamics, Durham, USA) and Mascot (v2.4, Matrix Science, Boston, MA) as previously described [29]. Search results were entered into Scaffold (v4.4.1.1; Proteome Software, Portland, OR) to determine protein identifications (80% peptide confidence; 95% protein confidence, with minimum of 2 unique peptides per protein), and imported back into Progenesis QI for relative quantitation. All proteomic data with accession IDs has been made available in the supplementary information.

### Serum MPA quantification

Serum MPA levels were quantified by mass spectrometry. Briefly, 85 μl of serum was spiked with 10 μl of 103 nM progesterone-D_9_ as an internal standard and incubated on ice for 1 hour. An ethyl acetate-hexane mixture (80:20) was then added (825 μl), vortexed, and then centrifuged at 300 x g for 5 min. The upper organic layer (600 μl) was then dried under a stream of nitrogen gas, reconstituted in a H_2_O: formic acid: MeOH (49:0.1:50) mixture, and profiled by LC-MS/MS using a using a Triple Quad Mass Spectrometer (ThermoFisher). Reference samples with known quantities of MPA, ranging from 0.01 ng/ml to 10 ng/ml, were used to construct a standard curve in each experiment. All values fell below the upper range of the curve. The lower limit of detection (LLOD) in this assay was 0.01 ng/ml (10 pg/ml) with calculated signal to noise (S/N) ratio of 3.53. Because we set an S/N ratio of 10 as optimal for a signal above background, we set the lower limit of quantitation (LOQ) at 0.05 ng/ml (50 pg/ml). A total of 443 women (66%) of women analyzed in this study had microbiome, host proteome and MPA levels from the sample time point analyzed. A total of 120 women were *Lactobacillus* dominant (LD) and had MPA levels >300 pg/ml (LD-High MPA), 140 were LD and had <299 pg/ml MPA detected (LD-Low MPA), 70 were non-LD and >300 pg/ml MPA detected and 113 were non-LD and had <299 pg/ml present in samples.

### Statistical analysis and modelling of human proteome data

Univariate analysis was performed to detect differences in protein levels associated with MPA levels within each microbiome group using two-tailed t tests. Significantly differentially expressed proteins passed a false discovery rate threshold of 5% (Benjamini-Hochberg method). Proteins significantly associated with MPA levels in LD women were analyzed by hierarchical cluster analysis (NMF package in r, v0.21.0) and distribution of participants within branches was assessed by two-tailed Fisher’s exact tests. Multivariate models were performed to determine a minimum protein signature to distinguish microbiome-MPA groupings and were constructed using the LASSO method for regression shrinkage and selection using glmnet in R (K-fold cross validation). PLSDA assessed the predictive ability of LASSO features to classify microbiome-MPA groups using 5-fold cross-validation repeated 50 times with 2 components selected after tuning, according to the MixOmics package in R (mixOmics v6.6.2).

### Logistic regression analysis

HIV infection risk among women using different hormonal contraceptives, stratified by major microbiome types was assessed using logistic regression. The distribution of epidemiological variables between women who reported using either DMPA or other hormonal contraceptives were assessed using univariate two-tailed Mann-Whitney U tests and Fisher’s exact tests where appropriate. Logistic regression models that included reported contraceptive method were used to estimate risk of HIV infection within each microbiome group (R version 3.5.1). Odds ratios (OR), 95% confidence intervals (CI) and two-sided *P*-values are reported. Models were adjusted for potential confounding variables, including baseline demographics such as age, study arm, income, education and HSV2 status, as well as partner number, sexual frequency, and condom use. Multivariate models were performed, which included all epidemiological variables found to be significantly different between groups of interest. A logistic regression model assessing the interaction between contraceptives used and microbiome type are reported with the corresponding two-sided interaction *P* value. Sensitivity analyses were performed to determine the robustness of major associations between microbiome status, contraceptive use, and HIV risk. As 8.3% of women changed contraceptive method between baseline and the sampled study visit, contraception status at baseline was assessed to determine if interactions with microbiome type remained significantly associated with HIV acquisition risk. Our previous study showed that tenofovir was less effective in non-*Lactobacillus* dominant women [36]. To control for the effect of Tenofovir, we repeated the stratified logistic regression analysis in women who were randomized to the placebo gel arm only.

## Supporting information

S1 Fig Vaginal microbiome composition in CAPRISA 004 participants. Mass spectrometry was used to identify bacterial proteins present in cervicovaginal mucus of 685 women who reported using depot medroxyprogesterone acetate (DMPA, n = 449), norethindrone/norethisterone enanthate (NET-EN, n = 123), a combined oral contraceptive pill (COC, n = 97), or no hormonal contraceptives (Non-HC, n = 16). A composition plot shows the proportion of microbial proteins assigned to each taxon for each sample, and the microbiome profiles identified, including *Lactobacillus* and non-*Lactobacillus* dominant microbiomes. The most abundant taxa at the genus level, in descending order, included *Lactobacillus* (59.02%), *Gardnerella* (21.64%), *Prevotella* (3.43%), *Pseudomonas* (2.73%) and *Mobiluncus* (2.15%) (S3 Table). *Lactobacillus-*dominant microbiomes could be further resolved into two distinct sub-groups, including those that were primarily composed of either *L*. *crispatus*, or *L*. *iners*, and a smaller group with no specific *Lactobacillus* species predominant. Two non-LD sub-groups could be identified; one that was dominated by *G*. *vaginalis* (median H index = 0.98), and the other with no specific dominant taxa that was highly diverse (median H index = 1.16). Hierarchical clustering was performed on bacterial proportion data using a Euclidean distance metric, and no grouping by hormonal contraceptive group was observed. DMPA = Depo-Medroxyprogesterone Acetate, NET-EN = Norethisterone enanthate, COC = Combined oral contraceptives, HC = Hormonal contraceptives.
(TIF)

S2 Fig Vaginal microbiome composition and diversity in women using hormonal contraceptives. Mass spectrometry was used to identify bacterial proteins present in cervicovaginal mucus of 685 women who reported using depot medroxyprogesterone acetate (DMPA, n = 449), norethindrone/norethisterone enanthate (NET-EN, n = 123), a combined oral contraceptive pill (COC, n = 97), or no hormonal contraceptives (Non-HC, n = 16). (A) A stacked bar chart shows the reported contraceptive frequency across the whole cohort, and among major microbiome groups (LD = *Lactobacillus* dominant, n = 407; Non-LD = Non-*Lactobacillus* dominant, n = 278). (B) A principal component (PC) analysis of women by taxa composition shows no clustering by hormonal contraceptive group. (C) The corresponding alpha-diversities (Shannon’s H Index) according to contraceptive type are displayed in violin plots. (D) Box-and-whisker plots of taxa levels between contraceptive groups are displayed for the most abundant taxa identified. Statistical differences based on Man Whitney U tests of bacterial proportions with FDR adjusted p values are displayed.
(TIF)

S3 Fig Bacterial taxa abundance between hormonal contraceptive groups. Volcano plots display statistical differences in relative bacterial abundance levels between reported contraceptives used at study visit (assessed by Man Whitney U tests). The log_10_ FDR adjusted p values and average % differences in microbial proportion are displayed. Tests were performed (A) across all women, (B) within *Lactobacillus*-dominant (LD) women and (C) within non-*Lactobacillus* dominant (non-LD) women.
(TIF)

S4 Fig Hierarchical cluster analysis of cervicovaginal inflammation pathways shows overlap between women with high MPA levels and non-*Lactobacillus* dominant vaginal microbiomes. (A) A heatmap shows patterns of cervicovaginal protein expression associated with serum MPA levels. LD/Low MPA individuals clustered independently to the left (branch 1—low immune activation), and LD/High MPA individuals clustered with non-LD women to the right (branch 2—high immune activation). Distinguishing molecular pathways are highlighted for each branch. (B) Stacked bar charts showing the proportion of women in each microbiome-MPA group who fell into the lower (Branch 1) or higher immune activation (Branch 2). Differences in proportions were calculated using two-tailed Fisher’s exact tests.
(TIF)

S5 Fig Confirmation of dose-dependent association between serum MPA levels and the vaginal proteome. The x axis shows the proteome differences between women who had no-low vs. med-high MPA levels by two-tailed *t*-test as presented in Fig 2. The y-axis shows MPA-protein correlations (Spearman’s rank correlation, SRC) to account for a gradient effect of MPA. Significant protein alterations with MPA use were seen in *Lactobacillus* dominant women by both *t*-test and correlation analyses. Minimal DMPA-proteome associations were detected by either analysis in non-*Lactobacillus* dominant women.
(TIF)

S6 Fig Protein expression of LASSO-selected features within microbiome-MPA groups. A LASSO model selected 17 features to distinguish women with *Lactobacillus* dominant microbiomes and low levels of MPA from other microbiome-MPA groups. Protein expression levels are plotted for each microbiome-MPA group in box and whisker plots for factors that were either (A) positively (B) negatively loaded within the model.
(TIF)

S7 Fig Host mucosal inflammation pathways associate with microbiome profiles and abundance of *Lactobacillus* species. (A) Degree of mucosal proteome differences between women with different vaginal microbiome profiles. Statistical significance thresholds of each protein are denoted by colour: FWER adj. *P*<0.05 (green), FDR adj. *P*<0.05 (burgundy), unadjusted *P*<0.05 (yellow), *P>*0.05 (ns, blue). (B) Activation scores of immune pathways significantly different between microbiome groups annotated using the Ingenuity Pathway Analysis database. Inflammatory and tissue remodeling pathways are primarily upregulated with non-*Lactobacillus*, *G*. *vaginalis-*dominant, and polymicrobial microbiomes. Pathways significantly associated (green) or trending at an association with (yellow) MPA levels in LD women are overlaid. LD: *Lactobacillus*-dominant women; non-LD: Non-*Lactobacillus*-dominant women; FWER; Family Wise Error Rate; FDR; False Discovery Rate; ns; Not Significant; NA: Normalized Abundance.
(TIF)

S8 Fig Functional microbiome pathway differences were not observed in *Lactobacillus* dominant women using different hormonal contraceptives. (A) Average functional microbiome composition pathway profiles for major microbiome groups and reported contraceptive groups across *Lactobacillus* dominant women. (B) A principal component (PC) plot of overall taxa composition based on total bacterial protein levels binned to the pathway (KEGG, KO-level) level shows no clustering of women by contraceptive reported at visit. (C) Forrest plots show bacterial KO pathways that were differentially abundant between *Lactobacillus* dominant women who reported using DMPA and those who reported using either NET-EN, COC or Non-HC based on above/below median levels of each pathway (Fisher’s Exact Test). Pathways were denoted as significant after Benjamini-Hochberg adjustment (red), significant at an unadjusted α<0.05 (grey), non-significant (black), or could not be assessed due to low pathway coverage (white).
(TIF)

S1 Table Clinical and demographic characteristics of study participants using different contraceptive methods in CAPRISA 004. (XLSX)

S2 Table Hormonal contraceptive rates upon study enrollment in CAPRISA 004. (XLSX)

S3 Table Bacterial taxa proportion represented in vaginal samples from study participants. (XLSX)

S4 Table Clinical, behavioural and demographic characteristics of CAPRISA 004 participants based on vaginal microbiome profiles. (XLSX)

S5 Table Probability of HIV seroconversion by microbiome type among women using different contraceptives at sample visit. (XLSX)

S6 Table Probability of HIV seroconversion by microbiome type among women using different contraceptives at study enrollment. (XLSX)

S7 Table Clinical, behavioural and demographic characteristics of CAPRISA 004 participants based on vaginal microbiome profiles and hormonal contraceptive type reported at sample visit. (XLSX)

S8 Table Adjusted logistic regression model estimates of risk associated with DMPA use relative to women on COC or NET-EN. (XLSX)

S9 Table Adjusted Cox proportional hazards estimates of risk associated with Non-Lactobacillus dominant women relative to Lactobacillus dominant women. (XLSX)

S10 Table Proportion of subjects with detectable MPA levels by contraceptive type reported at visit. (XLSX)

S11 Table Taxa level differences by MPA levels. (XLSX)

S1 Data All proteome data and MPA results. (XLSX)
