# Supplementary figures and images for: Vaginal microbiome-hormonal contraceptive interactions associate with the mucosal proteome and HIV acquisition
Source: PLoS Pathog. 2020 Dec 23;16(12):e1009097. doi: 10.1371/journal.ppat.1009097 (PMC7790405; doi:10.1371/journal.ppat.1009097)

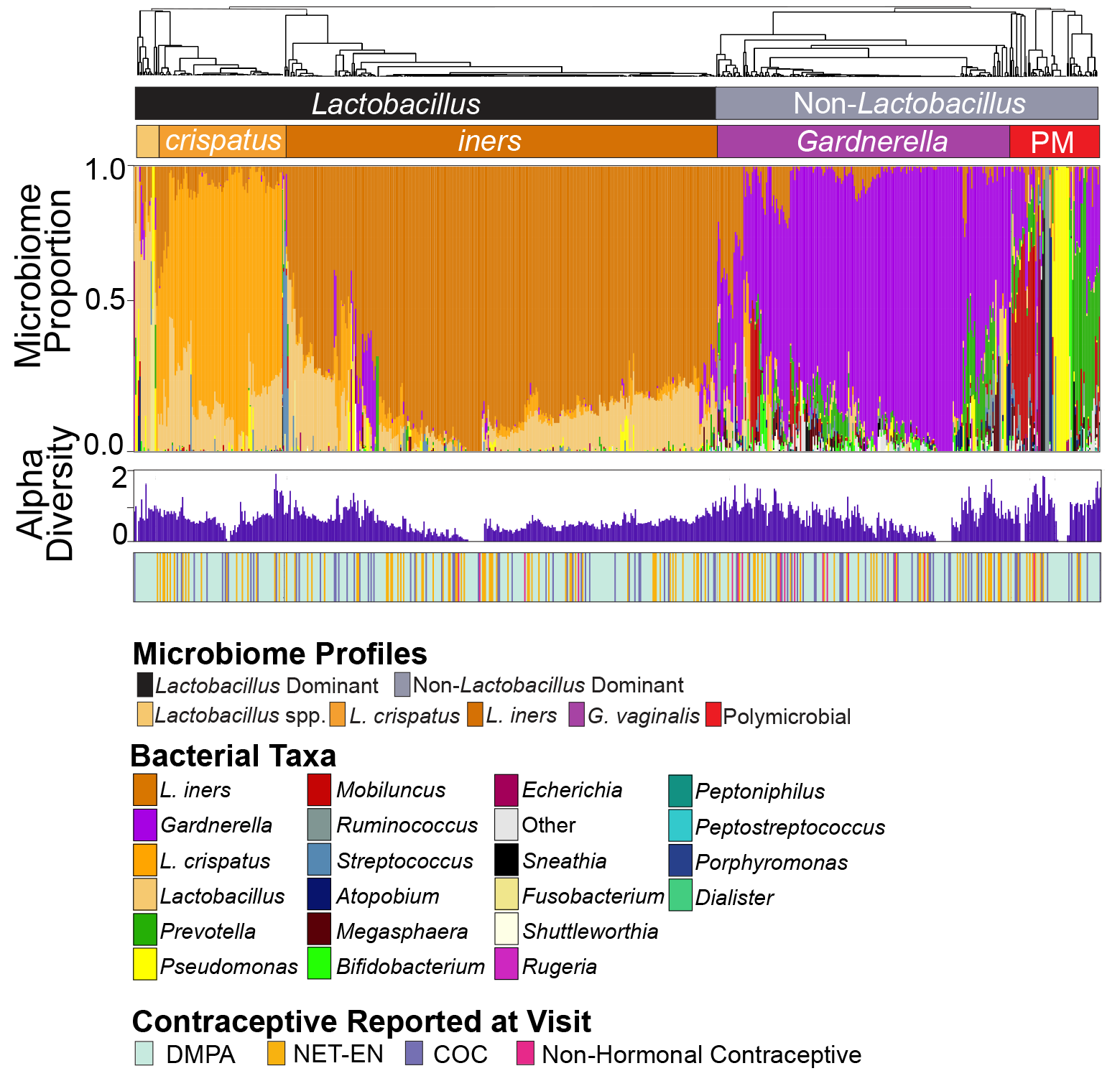

Supplement: S1 Fig — Mass spectrometry was used to identify bacterial proteins present in cervicovaginal mucus of 685 women who reported using depot medroxyprogesterone acetate (DMPA, n = 449), norethindrone/norethisterone enanthate (NET-EN, n = 123), a combined oral contraceptive pill (COC, n = 97), or no hormonal contraceptives (Non-HC, n = 16). A composition plot shows the proportion of microbial proteins assigned to each taxon for each sample, and the microbiome profiles identified, including Lactobacillus and non-Lactobacillus dominant microbiomes. The most abundant taxa at the genus level, in descending order, included Lactobacillus (59.02%), Gardnerella (21.64%), Prevotella (3.43%), Pseudomonas (2.73%) and Mobiluncus (2.15%) (S3 Table). Lactobacillus-dominant microbiomes could be further resolved into two distinct sub-groups, including those that were primarily composed of either L. crispatus, or L. iners, and a smaller group with no specific Lactobacillus species predominant. Two non-LD sub-groups could be identified; one that was dominated by G. vaginalis (median H index = 0.98), and the other with no specific dominant taxa that was highly diverse (median H index = 1.16). Hierarchical clustering was performed on bacterial proportion data using a Euclidean distance metric, and no grouping by hormonal contraceptive group was observed. DMPA = Depo-Medroxyprogesterone Acetate, NET-EN = Norethisterone enanthate, COC = Combined oral contraceptives, HC = Hormonal contraceptives. (TIF) [file ppat.1009097.s001.tif]

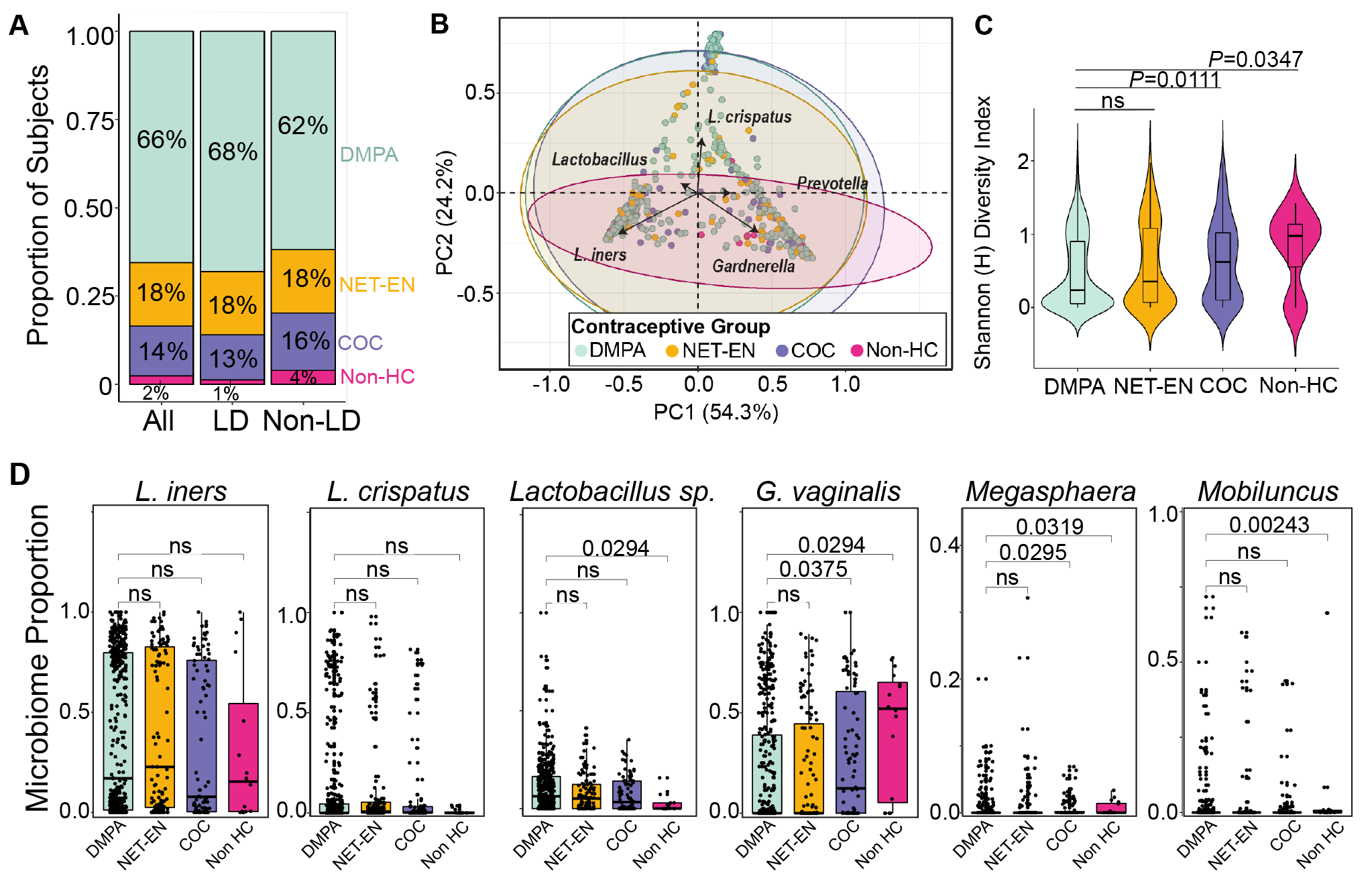

Supplement: S2 Fig — Mass spectrometry was used to identify bacterial proteins present in cervicovaginal mucus of 685 women who reported using depot medroxyprogesterone acetate (DMPA, n = 449), norethindrone/norethisterone enanthate (NET-EN, n = 123), a combined oral contraceptive pill (COC, n = 97), or no hormonal contraceptives (Non-HC, n = 16). (A) A stacked bar chart shows the reported contraceptive frequency across the whole cohort, and among major microbiome groups (LD = Lactobacillus dominant, n = 407; Non-LD = Non-Lactobacillus dominant, n = 278). (B) A principal component (PC) analysis of women by taxa composition shows no clustering by hormonal contraceptive group. (C) The corresponding alpha-diversities (Shannon’s H Index) according to contraceptive type are displayed in violin plots. (D) Box-and-whisker plots of taxa levels between contraceptive groups are displayed for the most abundant taxa identified. Statistical differences based on Man Whitney U tests of bacterial proportions with FDR adjusted p values are displayed. (TIF) [file ppat.1009097.s002.tif]

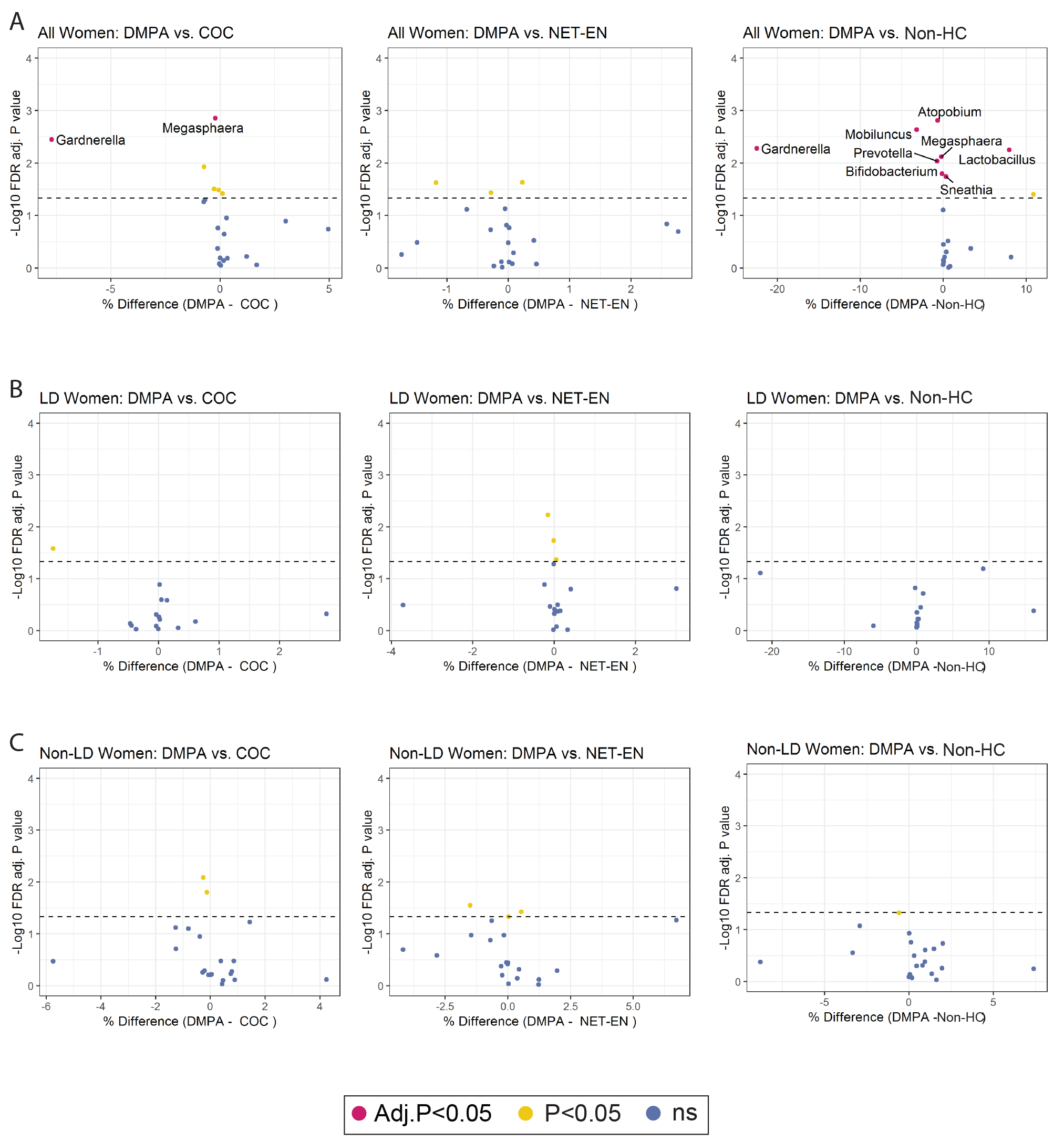

Supplement: S3 Fig — Volcano plots display statistical differences in relative bacterial abundance levels between reported contraceptives used at study visit (assessed by Man Whitney U tests). The log10 FDR adjusted p values and average % differences in microbial proportion are displayed. Tests were performed (A) across all women, (B) within Lactobacillus-dominant (LD) women and (C) within non-Lactobacillus dominant (non-LD) women. (TIF) [file ppat.1009097.s003.tif]

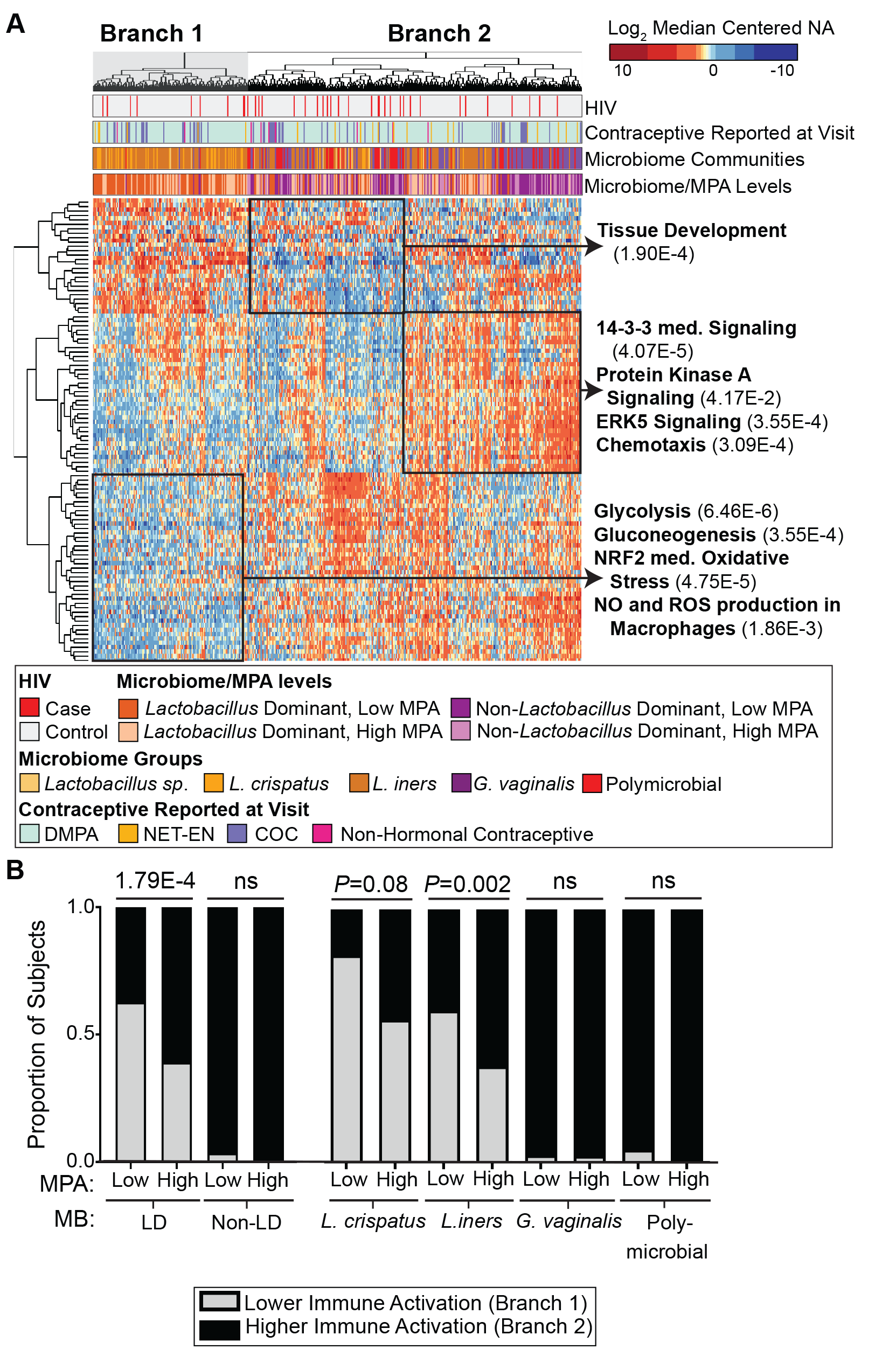

Supplement: S4 Fig — (A) A heatmap shows patterns of cervicovaginal protein expression associated with serum MPA levels. LD/Low MPA individuals clustered independently to the left (branch 1—low immune activation), and LD/High MPA individuals clustered with non-LD women to the right (branch 2—high immune activation). Distinguishing molecular pathways are highlighted for each branch. (B) Stacked bar charts showing the proportion of women in each microbiome-MPA group who fell into the lower (Branch 1) or higher immune activation (Branch 2). Differences in proportions were calculated using two-tailed Fisher’s exact tests. (TIF) [file ppat.1009097.s004.tif]

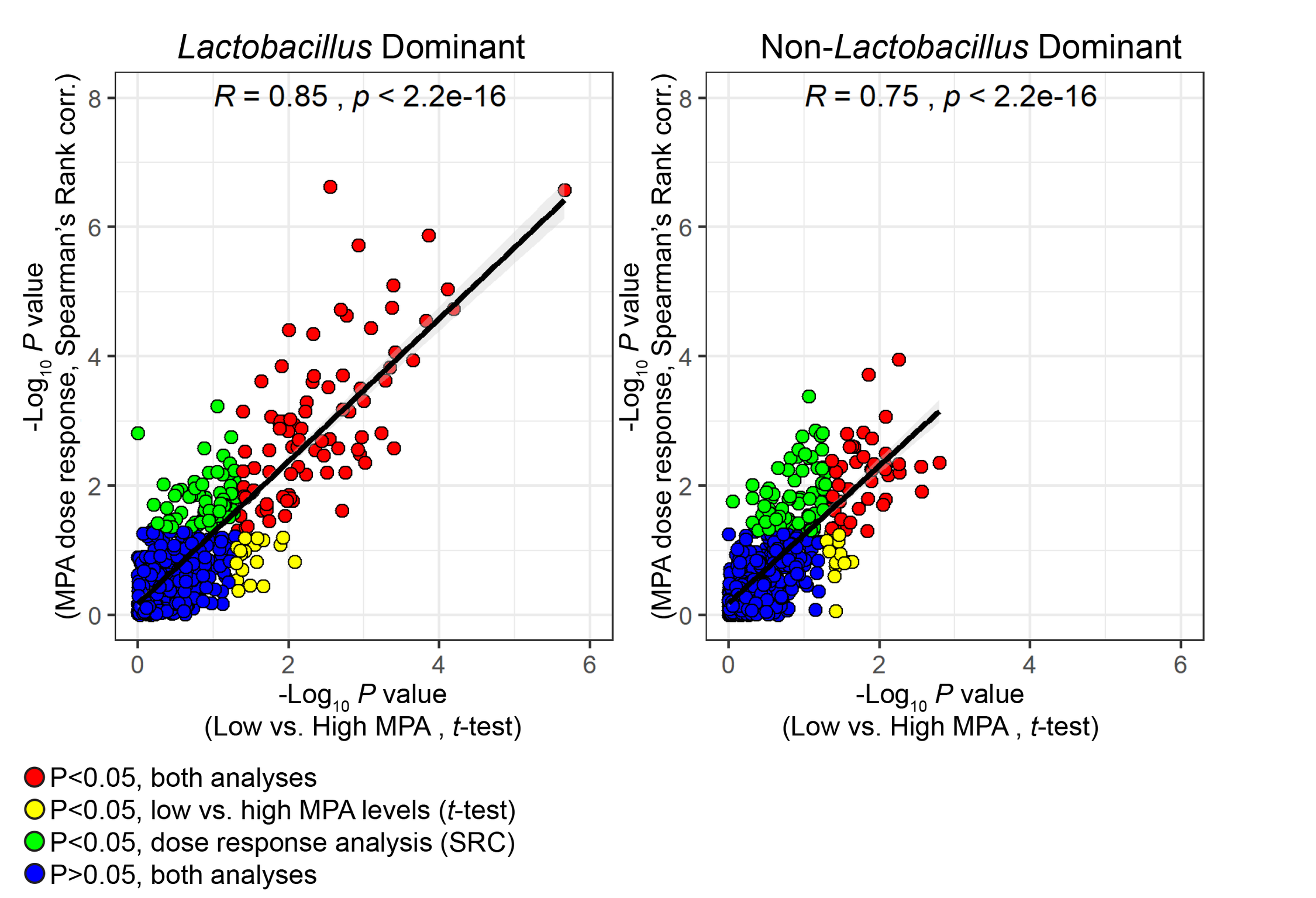

Supplement: S5 Fig — The x axis shows the proteome differences between women who had no-low vs. med-high MPA levels by two-tailed t-test as presented in Fig 2. The y-axis shows MPA-protein correlations (Spearman’s rank correlation, SRC) to account for a gradient effect of MPA. Significant protein alterations with MPA use were seen in Lactobacillus dominant women by both t-test and correlation analyses. Minimal DMPA-proteome associations were detected by either analysis in non-Lactobacillus dominant women. (TIF) [file ppat.1009097.s005.tif]

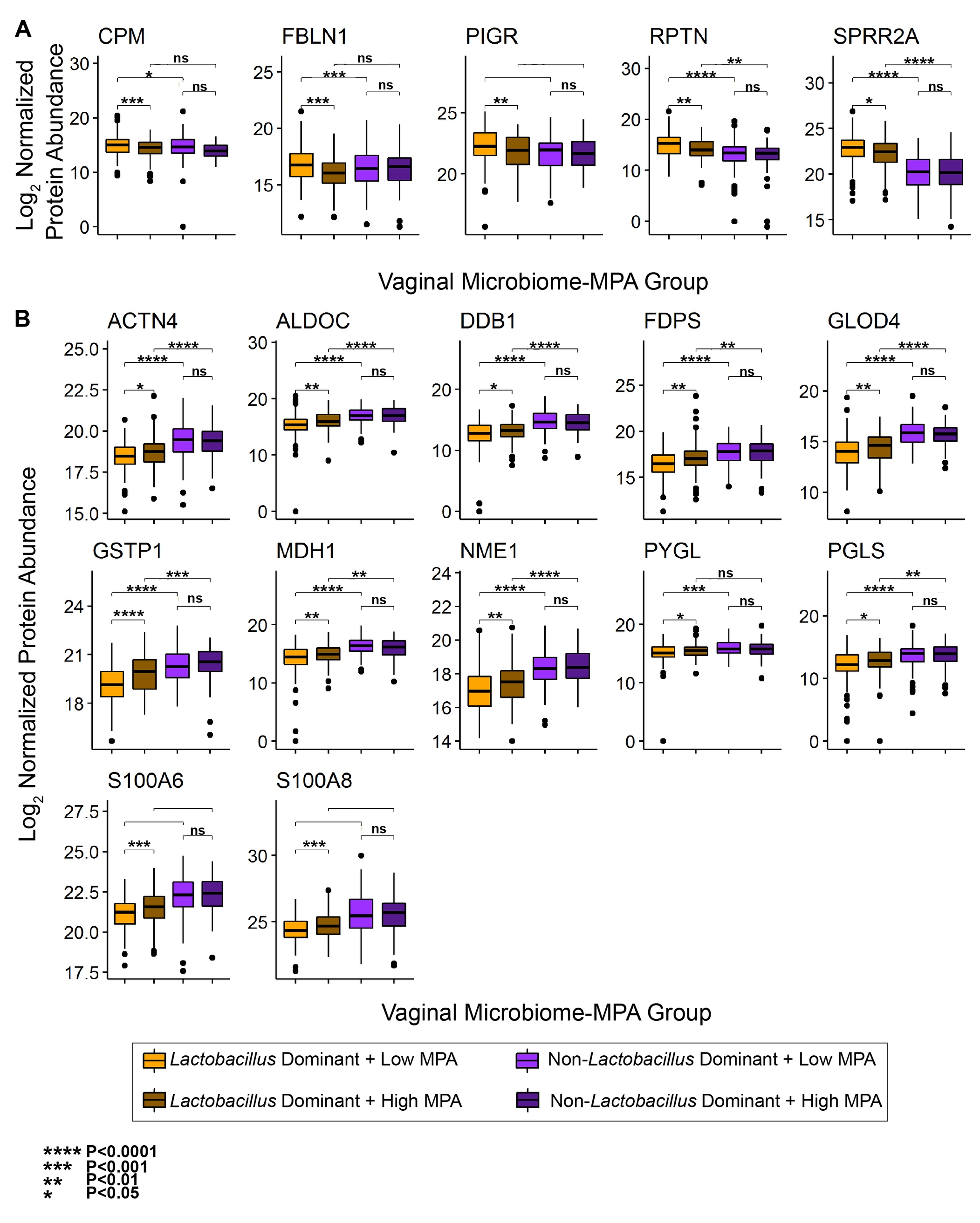

Supplement: S6 Fig — A LASSO model selected 17 features to distinguish women with Lactobacillus dominant microbiomes and low levels of MPA from other microbiome-MPA groups. Protein expression levels are plotted for each microbiome-MPA group in box and whisker plots for factors that were either (A) positively (B) negatively loaded within the model. (TIF) [file ppat.1009097.s006.tif]

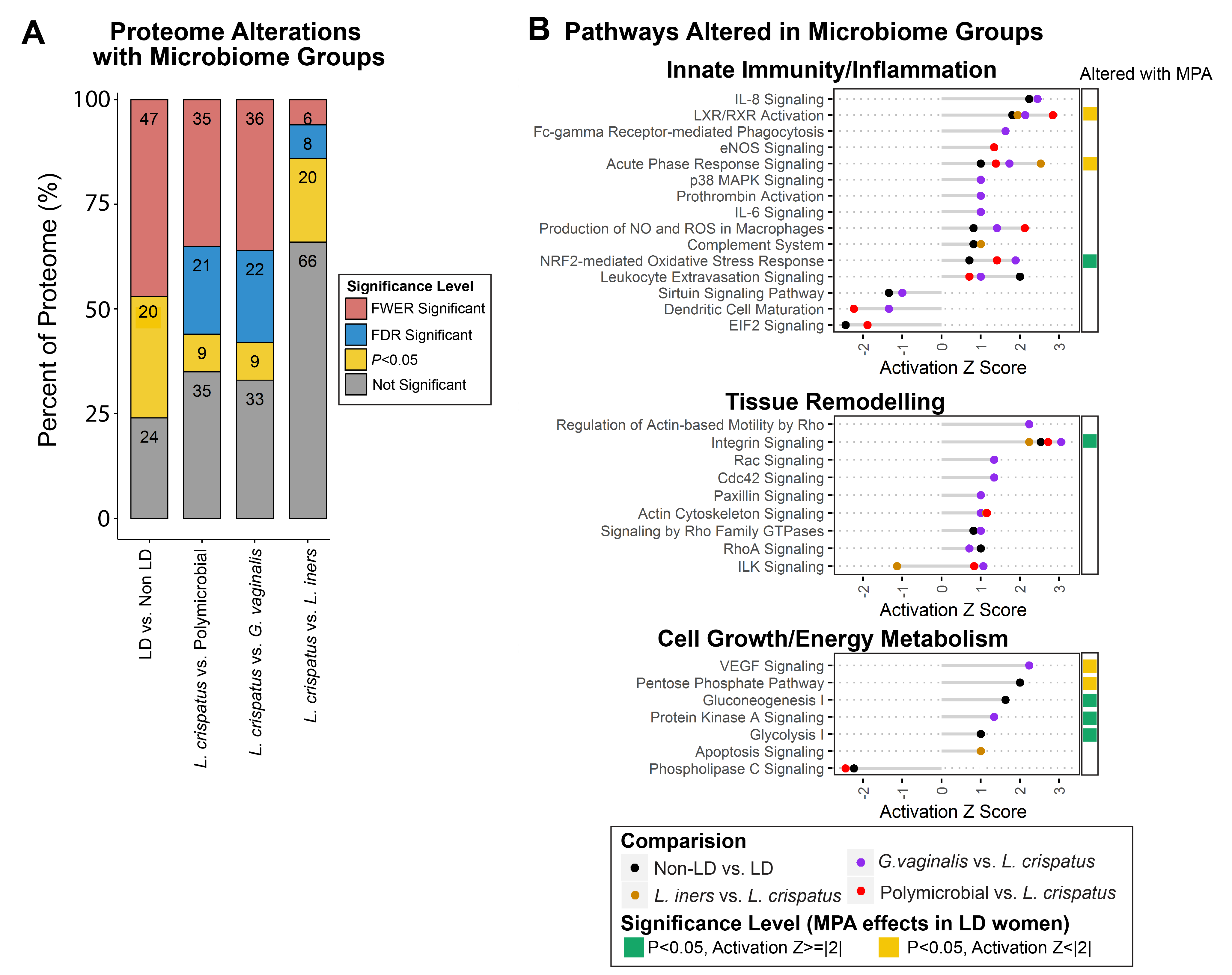

Supplement: S7 Fig — (A) Degree of mucosal proteome differences between women with different vaginal microbiome profiles. Statistical significance thresholds of each protein are denoted by colour: FWER adj. P<0.05 (green), FDR adj. P<0.05 (burgundy), unadjusted P<0.05 (yellow), P>0.05 (ns, blue). (B) Activation scores of immune pathways significantly different between microbiome groups annotated using the Ingenuity Pathway Analysis database. Inflammatory and tissue remodeling pathways are primarily upregulated with non-Lactobacillus, G. vaginalis-dominant, and polymicrobial microbiomes. Pathways significantly associated (green) or trending at an association with (yellow) MPA levels in LD women are overlaid. LD: Lactobacillus-dominant women; non-LD: Non-Lactobacillus-dominant women; FWER; Family Wise Error Rate; FDR; False Discovery Rate; ns; Not Significant; NA: Normalized Abundance. (TIF) [file ppat.1009097.s007.tif]

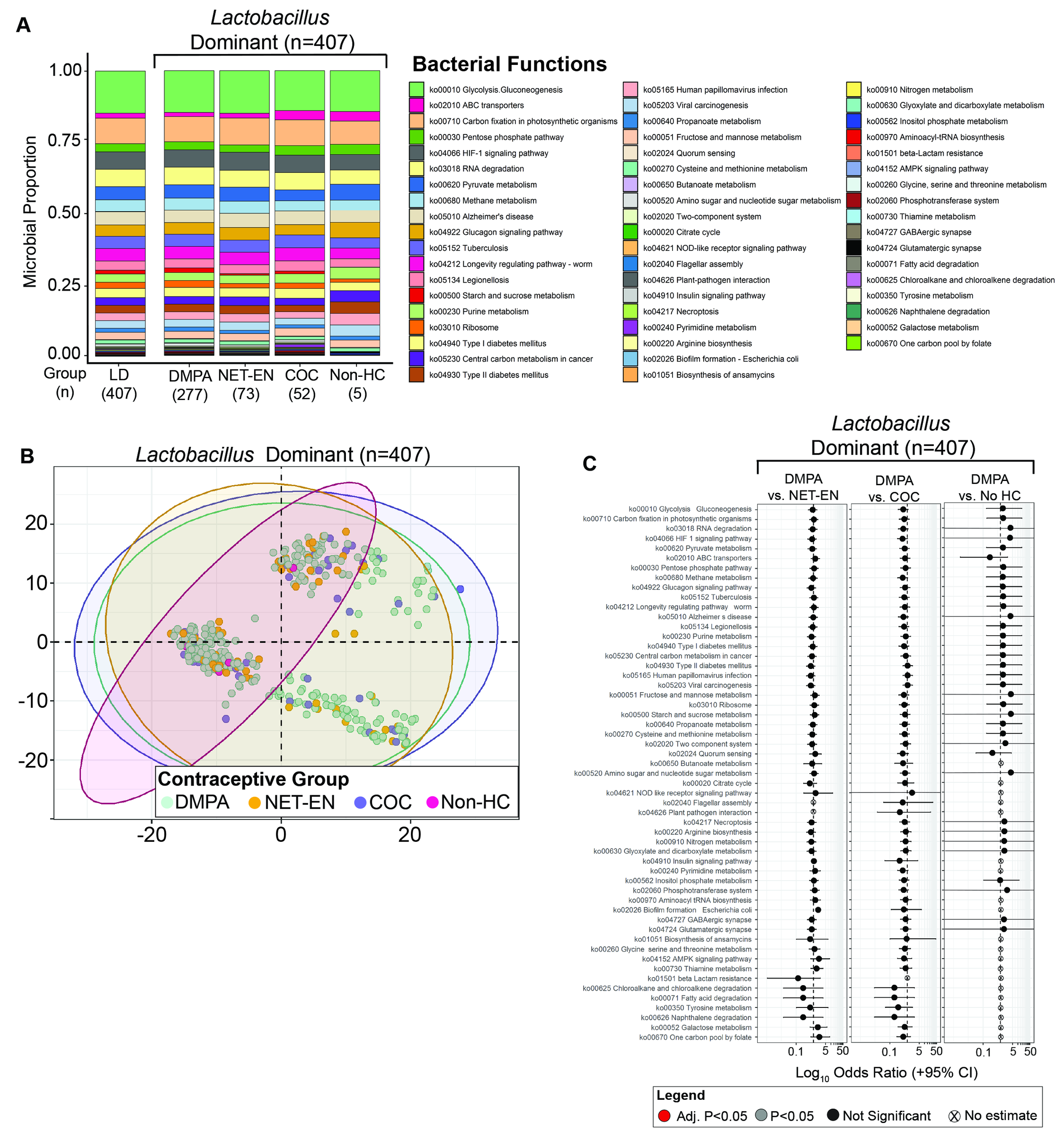

Supplement: S8 Fig — (A) Average functional microbiome composition pathway profiles for major microbiome groups and reported contraceptive groups across Lactobacillus dominant women. (B) A principal component (PC) plot of overall taxa composition based on total bacterial protein levels binned to the pathway (KEGG, KO-level) level shows no clustering of women by contraceptive reported at visit. (C) Forrest plots show bacterial KO pathways that were differentially abundant between Lactobacillus dominant women who reported using DMPA and those who reported using either NET-EN, COC or Non-HC based on above/below median levels of each pathway (Fisher’s Exact Test). Pathways were denoted as significant after Benjamini-Hochberg adjustment (red), significant at an unadjusted α<0.05 (grey), non-significant (black), or could not be assessed due to low pathway coverage (white). (TIF) [file ppat.1009097.s008.tif]
